# Tunable Polarization Conversion and Rotation based on a Reconfigurable Metasurface

**DOI:** 10.1038/s41598-017-11953-z

**Published:** 2017-09-21

**Authors:** M. Zhang, W. Zhang, A. Q. Liu, F. C. Li, C. F. Lan

**Affiliations:** 1 School of Energy Science and Engineering, Harbin Institute of Technology, Harbin, 150001 China; 2 School of Engineering and Applied Science, Harvard University, Cambridge, 02138 USA; 3 School of Electrical and Electronic Engineering, Nanyang Technological University, Singapore, 639798 Singapore; 4 School of Electrical and Electronic Engineering, Harbin University of Science and Technology, Harbin, 150080 China

## Abstract

Polarization is an important property of electromagnetic (EM) wave and different polarization manipulations are required for varied optical applications. Here we report a reconfigurable metasurface which achieves both the polarization conversion and the polarization rotation in THz regime. The metasurface is reconfigured through the micro-electro-mechanical-systems (MEMS) actuation. The cross polarization transmittance from a linear polarized incidence is experimentally tuned from 0 to 28% at 2.66 THz. In addition, the polarization rotation angle is effectively changed from −12.8° to 13.1° at 1.78 THz. The tunable bi-functional metasurface for polarization conversion and the polarization rotation can be flexibly applied in various applications such as imaging, polarization microscopy and material analysis, etc.

## Introduction

As an intrinsic property of the electromagnetic (EM) wave, polarization plays an important role in varied areas like optical imaging^1^, life science microscopy^2^ and multiplexed optical communication^3^, etc. The polarization direction can be effectively manipulated through the polarization conversion or the polarization rotation^4, 5^. The polarization conversion is conventionally achieved through anisotropic materials or structures^6^, while the polarization rotation usually relies on the Faraday Effect^7^. Gratings^8^ and photonic crystals^9^ were also proposed for the polarization direction control. In recent years, metasurface^10–12^ has been proposed as an effective means to manipulate the amplitude, the phase delay, and the polarization of the EM waves. The manipulation is highly dependent on the geometric structures of the metamolecules of the metasurface. Through proper metamolecule design, promising applications were realized including beam steering^13, 14^, diffraction limited focusing^15, 16^ and holographic imaging^17, 18^, etc.

The polarization conversion through a metasurface largely relies on the anisotropy of the metamolecule, while the polarization rotation is commonly realized through a chiral structured metamolecule^19–22^. For effective control of the polarization in real applications, flexible and multi-functional metasurfaces are proposed^23, 24^. Here, we report a bi-functional metasurface realizing both the polarization conversion and the polarization rotation in THz regime. The metasurface is designed to be reconfigurable through the MEMS actuation^25–27^. As a result, the polarization conversion efficiency and the polarization rotation angle are tuned. The cross transmittance of the linear polarization to its orthogonal direction is actively varied between 0 and 28%, while the polarization rotation angle is significantly tuned from −12.8° to 13.1° experimentally. The polarized THz wave could play an important role in THz communication multiplexing, anisotropic or birefringent material analysis and holographic imaging, etc.

## Results and Discussions

### Design of the tunable metasurface

**Figure 1:**
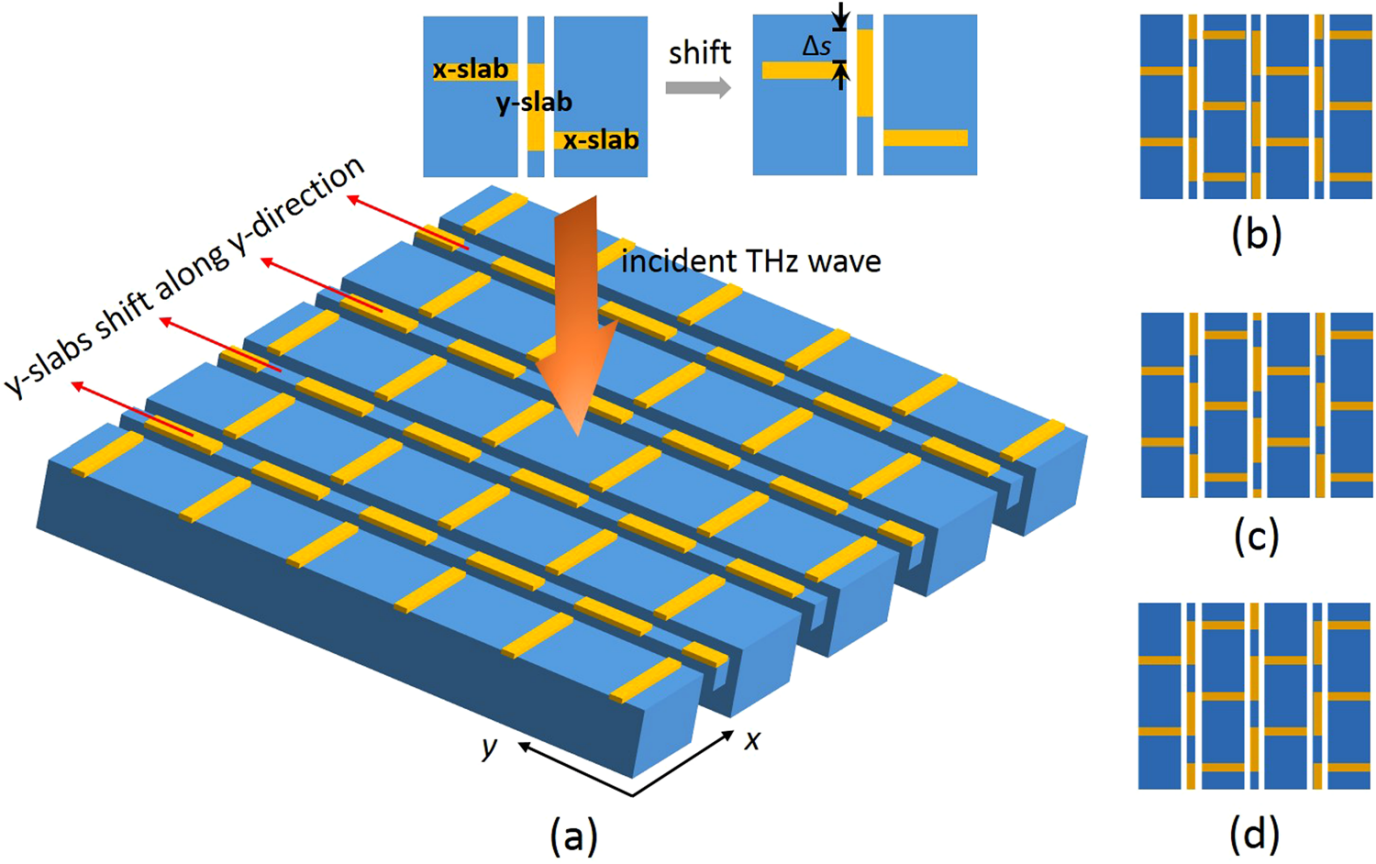
**(a)** The reconfigurable metasurface; **(b)** the “DOWN” state of the metasurface; **(c)** the “T” state of the metasurface; **(d)** the “UP” state of the metasurface.

The design of the tunable metasurface for the polarization conversion and the polarization rotation is illustrated in Fig. 1(a). The metamolecule in the metasurface consists of two kinds of metal slabs called the x-slabs and the y-slabs as indicated in Fig. 1(a). The x-slab is along the *x*-direction and has a length *L*_x_ = 30 µm and width *w*_x_ = 6 µm; the y-slab is along the *y*-direction and has a length *L*_y_ = 31 µm and the width *w*_y_ = 6 µm. While the x-slab is on a rigid substrate, the y-slab is patterned on a suspended beam and can be shifted by a micromachined actuator along the *y-*direction. As shown in Fig. 1(b), initially the left side of an x*-*slab is close to the top of a y-slab, and the right side of the x*-*slab is close to the bottom of another y-slab. This is like a descending stair structure and is named the “DOWN” state of the metasurface. In this state the metasurface is a planar chiral structure. The metamolecule is reconfigured to a “T” structure when all y-slabs are moved along the *y-*direction with a displacement Δ*s* of (*L*_y_ − *w*_y_)/2 = 12.5 µm as shown in Fig. 1(c). The metasurface now turns into a symmetrical achiral structure and is name the “T” state of the metasurface. The metasurface is further reconfigured to an ascending stair structure as illustrated in Fig. 1(d) when the y-slab is shifted with a total displacement Δ*s* of (*L*_y_ − *w*_y_) = 25 µm. This is defined as the “UP” state of the metasurface. In this state, the metamolecule is a mirror image of that in the “DOWN” state. The lattice constant, *a*_x_ and *a*_y_, is 80 µm and 50 µm, respectively. The *a*_y_ is set twice the length of the total displacement (*L*_y_ − *w*_y_) so that the metamolecule can be consistently transformed from one state to another during the y-slab shifting.

### Calculation results and discussions

**Figure 2:**
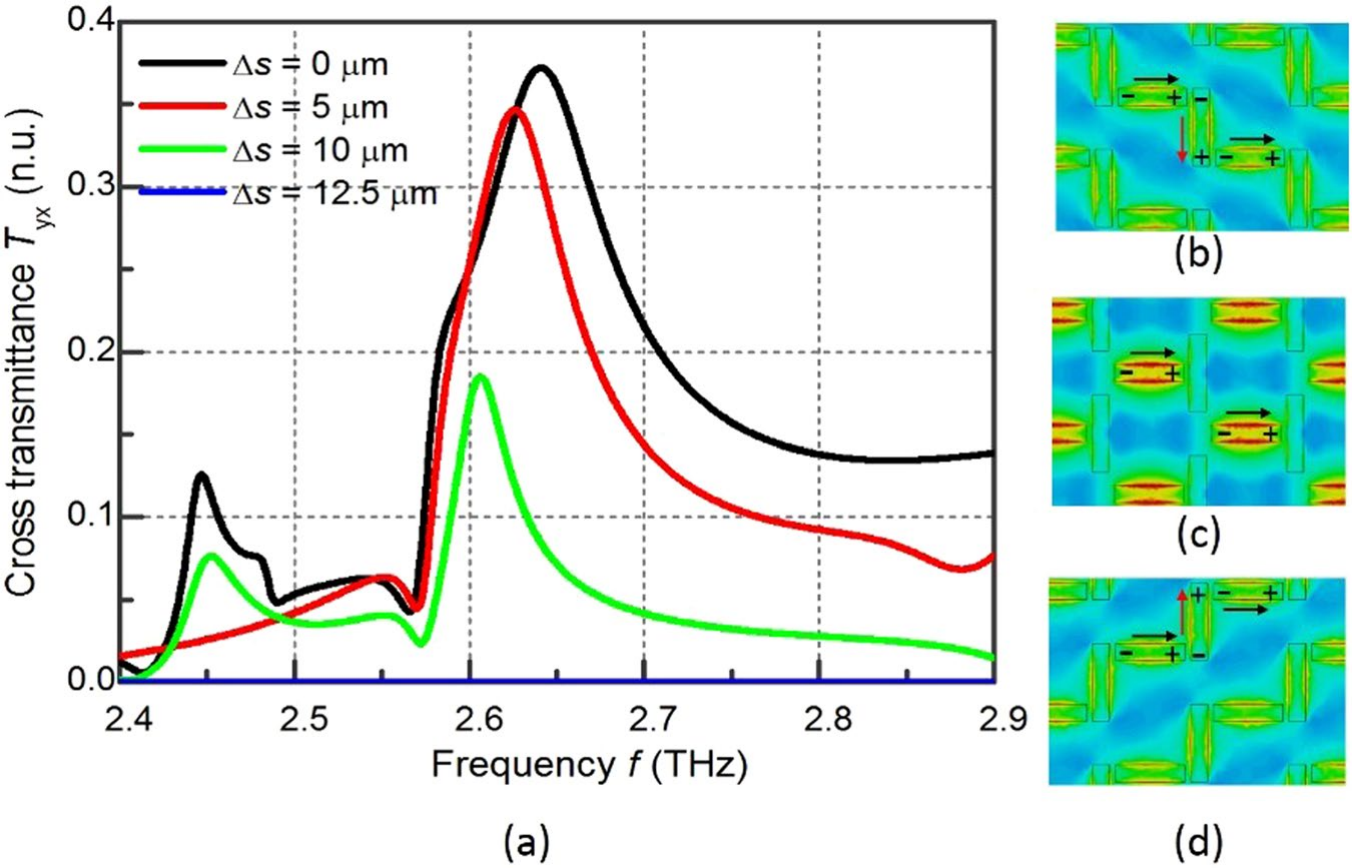
**(a)** The numerically calculated transmittance *T*_yx_ through the metasurface; **(b–d)** the calculated magnetic field intensity of the “DOWN” state, the “T” state and the “UP” state, respectively. The black arrows show the current induced directly by the incident electric field. The red arrows indicate the current induced through the coupling between the x-slabs and the y-slabs.

The conversion of the linear polarization to its orthogonal direction through the metasurface is firstly discussed. An x-polarized THz wave is normally incident on the metasurface and the cross polarized transmittance *T*_yx_ is numerically calculated. As shown in Fig. 2(a), in the “DOWN” state when Δ*s* = 0, *T*_yx_ has a peak value of 37% at 2.64 THz. As the y-slabs shift by 5 µm and 10 µm, the peak value drops to 34% and 18%, respectively. *T*_yx_ in the whole spectrum vanishes when Δ*s* increases to 12.5 µm and the metasurface changes to the “T” state. As the y-slabs further shift in the y-direction, *T*_yx_ starts to increase and comes back to 37% when Δs is 25 µm and the metasurface changes to the “UP” state. The *T*_yx_ is the same for a shifting of ∆*s*_0_ and a shifting of (25 µm − ∆*s*_0_) because the two states are the mirror image of each other and the couplings between the x-slab and the y-slab are the same.

The origin of the polarization conversion can be explained by investigating the induced magnetic field and the surface current on the metasurface at 2.64 THz under the normal incidence of the x-polarized THz wave as shown in Fig. 2(b–d) for the “DOWN” state, the “T” state, and the “UP” state, correspondingly. In the “DOWN” state, the surface current on the x-slab is directly induced by the incident electric field and is denoted with black arrows. Opposite electric charges are thus induced on the two ends of the y-slabs and the oscillation current is formed and is denoted with red arrows. In the “T” state, the surface current is mainly concentrating on the x-slab with little current on the y-slab due to weak couplings between x-slabs and y-slabs. Therefore, there is no conversion from the *x-*polarization to the *y-*polarization. In the “UP” state, the surface current excitation and coupling are the same as that in the “DOWN” state, except that the coupling induced current on the y-slab flows in the opposite direction.

**Figure 3:**
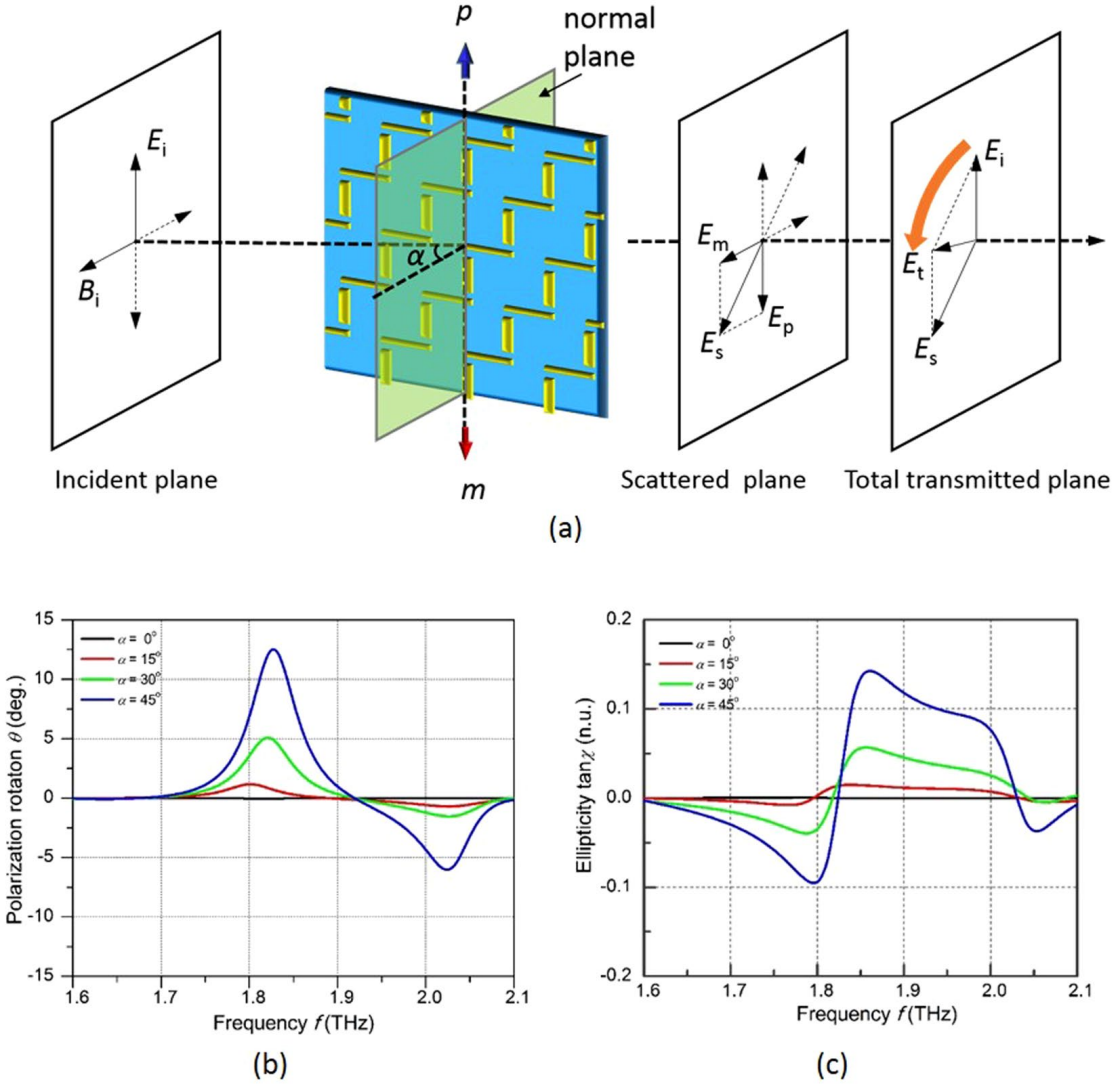
**(a)** The mechanism of the polarization rotation through the metasurface under oblique incidence **(b)** the polarization rotation angle and **(c)** the dichroism in the “DOWN” state with different incident angle.

As a 2D chiral structure, the metamolecule does not possess intrinsic chirality^28^. However, the extrinsic chirality of the metasurface can be realized under oblique incidence because the k-vector of the incident wave is not in the normal direction of the 2D chiral surface^29^. This can be further explained by the coupling between the induced electrical dipole and the magnetic dipole as shown in Fig. 3(a) when the EM wave is incident on the metasurface with an angle of *α*. An electric dipole ***p*** is excited in the metamolecule under the incident electric field and emits an electric field ***E***_p_. Meanwhile, a magnetic dipole ***m*** is also induced on the planar chiral structure due to oblique incidence. The ***m*** is either parallel or antiparallel with the electric dipole ***p*** and reradiates EM waves with electric field ***E***_m_. The combination of the ***E***_p_ and ***E***_m_ is the total scattering field ***E***_s_, which is not parallel to the incident ***E***_i_ due to the non-zero ***E***_m_. Therefore, the direction of the transmitted field ***E***_t_, which equals to “***E***_i_ + ***E***_s_” is rotated relative to the original ***E***_i_ direction.

The polarization rotation angle *θ* and the circular dichroism tan(*χ*), which is defined as the relative difference of the circularly polarized transmittance, can be expressed as in the Eqns (1) and (2) ^30^

1 $$\theta =({\phi }_{++}-{\phi }_{--})/2$$

and

2 $$\tan \,{\rm{\chi }}=\,({t}_{++}-{t}_{--})/({t}_{++}+{t}_{--})$$

where *t*_++_ (*t*_−−_) and *φ*_++_ (*φ*_−−_) are the transmittance and phase delay of the transmitted left (right) circularly polarized light from a left (right) polarized incident light, respectively. Figure 3(b) and (c) show the numerically calculated polarization rotation and dichroism of the THz wave passing through the metasurface in the “DOWN” state under different incident angles. The polarization rotation and the dichroism are 0 when *α* = 0° in the entire investigated frequency band, which confirms that there is no chirality in the planar metasurface under the normal incidence. As the incident angle increases, the absolute value *θ* and the tan(*χ*) become non-zero, which indicates a polarization rotation and a circular birefringence. The polarization rotation *θ* is increased up to 13° at 1.82 THz when *α* increases to 45°, which is an anti-clockwise polarization rotation.

**Figure 4:**
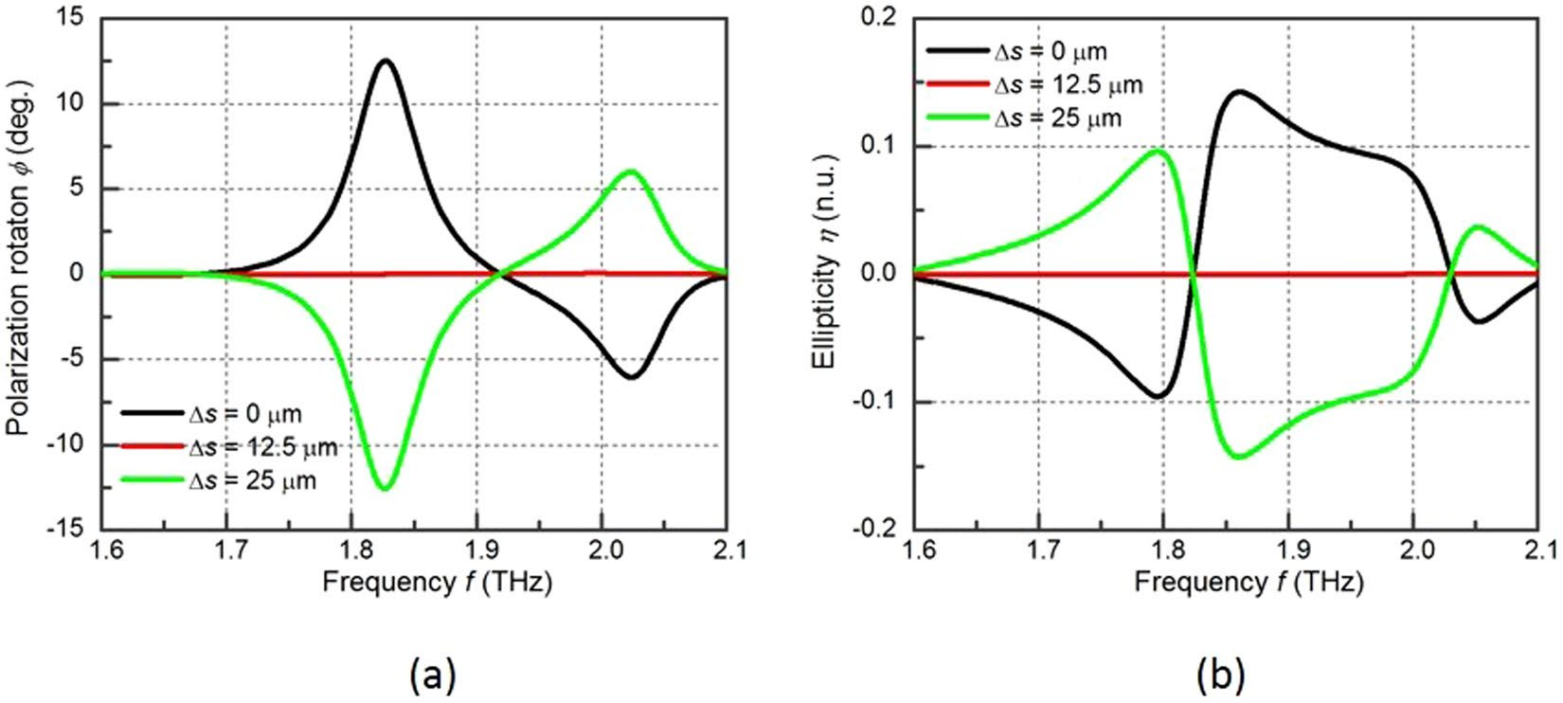
(**a**) The polarization rotation angle and **(b)** the dichroism of the metasurface at different y-slab displacements under 45° incident angle *α*.

The polarization rotation tunability is investigated through reconfiguring the metasurface from the “DOWN” state to the “UP” state as shown in Fig. 4(a) and (b) with *α* fixed at 45°. The polarization rotation decreases from 13° to 0° at 1.82 THz when the metasurface is tuned from the “DOWN” state to the “T” state. In fact the polarization rotation is 0 in the whole investigated spectrum, because the metasurface in the “T” state is a planar symmetric structure and no extrinsic chirality can be induced. A negative polarization rotation is observed between 1.75 THz and 1.90 THz when the metasurface is tuned to the “UP” sate with the ∆*s* of 25 µm. Comparing the polarization rotation angle in the “DOWN” state with that in the “UP” state, they have the same angle value with opposite sign. This is because the coupling direction between the electrical field and the magnetic field is reversed when reconfiguring the planar chiral metamolecule to its mirror image structure. Therefore, the polarization rotation is reversed.

### Experimental results and discussions

**Figure 5:**
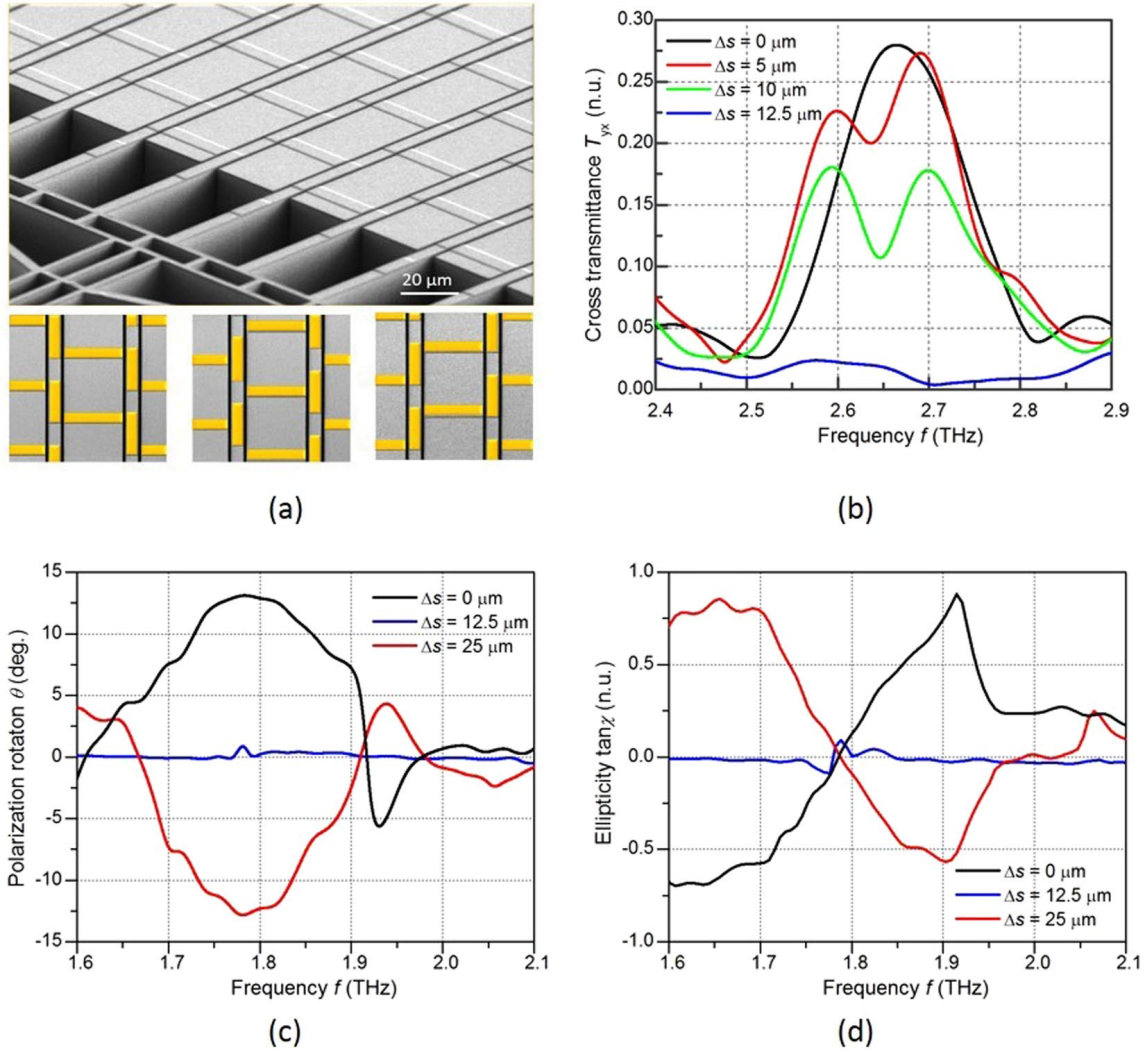
**(a)** SEM graph of the reconfigurable metasurface, the scale bar is 20 µm (top); the “DOWN” state (bottom left), the “T” state (bottom middle) and the “UP” state (bottom right) of the metasurface. **(b)** The measured transmittance *T*_yx_ through the metasurface at different y-slab shift distances under normal incidence; **(c)** the polarization rotation and **(d)** the dichroism of the THz wave through the metasurface in the different tuning states under incidence angle of 45°.

Figure 5(a) shows the scanning electron microscopic (SEM) image of the reconfigurable metasurface, which is fabricated using a MEMS process. The sample consists of a 200 × 125 element array which has a period of 50 μm × 80 μm in *x-y* plane. The x-slab and the y-slab in the metasurface are patterned on the fixed silicon bulk and movable silicon beam, respectively. The gap between the x-slab and the y-slab is 2 μm. The relative position of the two types of slabs is tunable when the slabs along *y*-direction are shifted through the integrated MEMS actuator. The tuning time for shifting the slabs for 25 µm is about 2 ms. The insertion of Fig. 5(a) shows the close-up view of the metasurface element in the “DOWN” state, the “T” state, and the “UP” state, respectively.

The cross transmittances of the x-polarized incident wave are measured when the element is tuned from the “DOWN” state to the “T” state as shown in Fig. 5(b). A high transmittance *T*_yx_ of 28% is obtained in the “UP” state at 2.66 THz. *T*_yx_ decreases as the x-slab shifts from 0 μm to 12.5 μm, which reconfigures the element from asymmetric structure to symmetric structure. As a result, the conversion from the x-polarized incident wave to the y-polarized transmitted wave decreases dramatically.

The measured polarization rotation and dichroism of the metasurface under 45° incidence are plotted in Fig. 5(c) and (d), respectively. A polarization rotation peak of 13.1° is observed at 1.78 THz in the “DOWN” state. The rotation decreases to almost 0 when the metamaterial is shifted to the “T” state, in which the metamolecule is a symmetric structure. The polarization rotation is then reversed to a negative value with a further shift of the y-slab. The rotation finally becomes −12.8° when the metasurface is tuned to the “UP” state. The reversed polarization rotation angle between the “DOWN” state and the “UP” state demonstrates the reversed coupling between the x-slab and the y-slab through the state switch.

## Conclusion

In conclusion, we have investigated theoretically and experimentally the polarization conversion and polarization rotations through the reconfigurable metasurface. It is demonstrated that significant tunability can be realized by changing the couplings between the metallic components in the metamolecule. The conversion efficiency is variable between 0 and 28% at 2.66 THz under normal incidence while the polarization rotation angle is effectively tunable from −12.8° to 13.1° with an incident angle of 45° at 1.78 THz. As a result, the tunability of the polarization conversion and the polarization rotation can be applied in many real time controlled applications such as the imaging and signal communications, etc.

## Methods

### Fabrication process

The fabrication of the reconfigurable metasurface starts with a silicon on insulator (SOI) wafer followed by aluminum metal patterning using physical vapor deposition (PVD) and optical lithography processes. The aluminum layer has two functions: one is to be patterned as the metamolecule structure and the other is to be used as the metal contact for the micromachined actuator. Deep reactive ion etching (DRIE) and SiO_2_ layer etching processes are then applied to suspend and drive the y-slab on the substrate.

### Experimental setup

A THz Time-domain spectroscopy (THz-TDS) Teraview spectra 3000 is used to measure the linearly polarized transmittance. This is converted to circularly polarized transmittance for polarization rotation determination. Electrical voltage is applied on a micromachined comb drive to move the y-slabs. The displacement can be expressed as Δ*x* = *KV* ^2^, where *K* is the actuation coefficient with the value of 0.04 in the design and depends on the structure size of the comb drive. A displacement ∆*x* of 12.5 µm and 25 µm are obtained from an actuation drive of 16 V and 26 V, respectively, in the measurement.
